# Braun anastomosis lowers the incidence of delayed gastric emptying following pancreaticoduodenectomy: a meta-analysis

**DOI:** 10.1186/s12876-018-0909-5

**Published:** 2018-11-26

**Authors:** Yanming Zhou, Bin Hu, Kongyuan Wei, Xiaoying Si

**Affiliations:** 1 Department of Hepatobiliary & Pancreatovascular Surgery, First affiliated Hospital of Xiamen University, Xiamen, China; 2 Department of Clinical Laboratory Medicine, First affiliated Hospital of Xiamen University, Xiamen, China; 3 Department of General Surgery, First Hospital of Lanzhou University, Lanzhou, China

**Keywords:** Delayed gastric emptying, Braun enteroenterostomy, Pancreatoduodenectomy, Meta-analysis

## Abstract

**Background:**

Delayed gastric emptying (DGE) is one of the most frequent complications following pancreaticoduodenectomy. This meta-analysis aimed to evaluate the impact of Braun enteroenterostomy on DGE following pancreaticoduodenectomy.

**Methods:**

A systematic review of the literature was performed to identify relevant studies. Statistical analysis was carried out using Review Manager software 5.3.

**Results:**

Eleven studies involving 1672 patients (1005 in Braun group and 667 in non-Braun group) were included in the meta-analysis. Braun enteroenterostomy was associated with a statistically significant reduction in overall DGE (odds ratios [OR] 0.32, 95% confidence intervals [CI] 0.24 to 0.43; *P* <0.001), clinically significant DGE (OR 0.27, 95% CI 0.15 to 0.51; *P* <0.001), bile leak (OR 0.50, 95% CI 0.29 to 0.86; *P* = 0.01), and length of hospital stay (weighted mean difference -1.66, 95% CI -2.95 to 00.37; *P* = 0.01).

**Conclusions:**

Braun enteroenterostomy minimizes the rate and severity of DGE following pancreaticoduodenectomy.

## Background

Delayed gastric emptying (DGE) is one of the most frequent morbidity following pancreaticoduodenectomy with the reported incidence of 14–61% [1]. Although DGE is not life-threatening, it may prolong the length of hospital stay and increase the medical cost. In addition, severe DGE may delay adjuvant therapies for patients with cancer.

To prevent DGE, Braun enteroenterostomy between the afferent and efferent limbs distal to the gastroenterostomy site was introduced and two meta-analyses of several non-randomized controlled trials (NRCTs) demonstrated that it could reduce the occurrence of DGE [2, 3]. However, recent three randomized controlled trials (RCTs) failed to confirm this finding [4–6]. It seems that controversies still exist concerning the effect of Braun enteroenterostomy on DGE. The aim of the present meta-analysis is to provide an updated evaluation on this issue.

## Methods

The study was conducted following the Preferred Reporting Items for Systematic Reviews and Meta- Analyses (PRISMA) [7]. The protocol of PRISMA consists of a 17-item checklist intended to facilitate the preparation and reporting of a robust protocol for the systematic review and meta- analyses that summarize aggregate data from studies, particularly the evaluations of the effects of interventions.

### Study selection

A comprehensive search was performed on electronic databases (PUBMED, EMBASE and Cochrane Library) from inception until May 2017 to identify relevant studies using the key words “pancreaticoduodenectomy,” “Braun enteroenterostomy” and “delayed gastric emptying.” Bibliography of retrieved papers was further manually searched for additional studies. Studies that evaluated the influence of Braun enteroenterostomy on DGE after pancreaticoduodenectomy were considered for inclusion. Non-English language articles, animal studies, abstracts, letters, proceedings from scientific meetings, editorials and expert opinions, duplicates, and noncomparative studies or case series were excluded.

### Outcome measures

The primary endpoint was the rate of patients with overall DGE and clinically relevant (grade B-C according to the International Study Group of Pancreatic Surgery [ISGPS] classification) DGE [8]. The secondary endpoints were operative details, clinical parameters related to DGE, other complications like pancreatic fistula, mortality and length of hospital stay.

### Data extraction

Two independent reviewers (ZY and SX) respectively assessed each eligible study. Disagreements were resolved by discussion. The standard information extracted from each included study was as follows: first author, year of publication, sample sizes, characteristics of the studies, and endpoints.

### Assessment of methodological quality

NRCTs were evaluated using Newcastle-Ottawa quality assessment scale [9]. The quality scale ranges from 0 to 9 stars, and studies with 6 stars or greater were considered to be of high quality. RCTs were scored using the Jadad composite scale [10]. The quality scale ranges from 0 to 5 points, and studies with 3 or more scores were considered to be of high quality.

### Statistical methods

Meta-analysis was carried out using Review Manager (RevMan) software 5.3 (Cochrane Collaboration). For categorical variables, statistical analysis was carried out using the weighted mean difference (WMD) with 95% confidence intervals (CI). For dichotomous variables, statistical analysis was carried out using the odds ratios (OR) with 95% CI. Heterogeneity was evaluated by* I*^2^ test. The random-effects model was used to analyze data if there was significant heterogeneity (*I*^2^ ≥ 50%) between studies; otherwise, the fixed-effects model was used. Risk for bias was evaluated using a funnel plot based on the result of DGE.

## Results

### Eligible studies

**Fig. 1:**
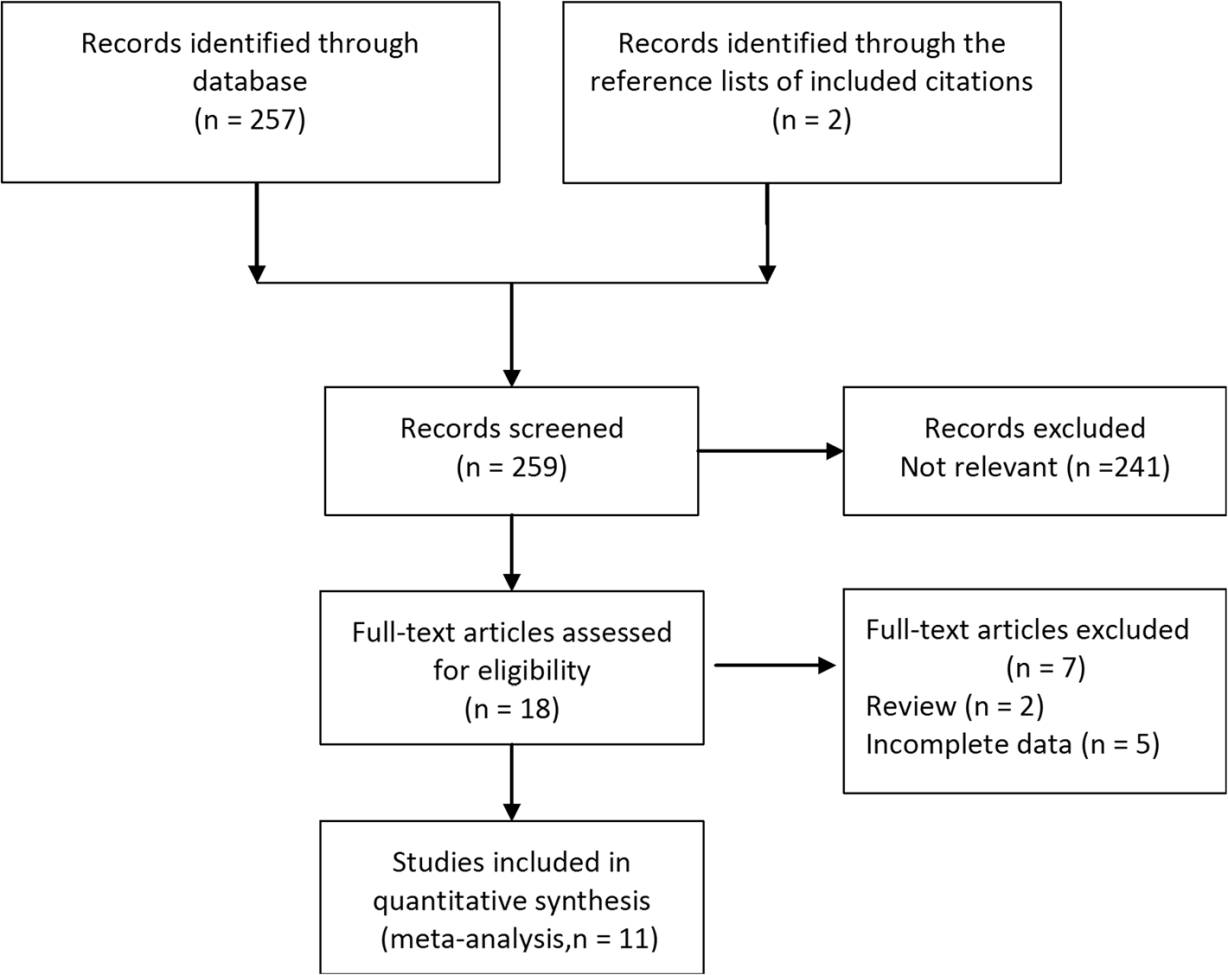
Study flow chart

The process of identifying eligible studies is shown in Fig. 1. The search strategy generated 11 articles [4–6, 11–18] that fulfilled the inclusion criteria with a total of 1672 patients (1005 in Braun group and 667 in non-Braun group). The characteristics of the included studies are shown in Table 1.

**Table 1:** Baseline characteristics of studies included in the meta-analysis

Reference	Year	Design	EI (Region)	N	BG (M/F, age^a^)	NBG (M/F, age^a^)	Score
Kakaei [4]	2015	RCT	2013–2013 (Iran)	30	14 (10/5, 57)	15 (10/5, 55)	2
Hwang [5]	2016	RCT	2013–2014 (Korea)	60	30 (19/11, 69)	30 (19/11, 63)	3
Fujieda [6]	2017	RCT	2011–2016 (Japan)	68	34 (20/14, 66)	34 (24/10, 72)	3
Hochwald [11]	2010	NRCT	2001–2006 (USA)	105	70 (−/−, 65)	35 (−/−, 4)	8
Nikfarjam [12]	2012	NRCT	2009–2011 (Australia)	44	24 (15/9, 67)	20 (14/6, 70)	7
Cordesmeye [13]	2014	NRCT	2004–2011 (German)	45	51 (27/24, −)	62 (32/30, −)	6
Wang [14]	2014	NRCT	2008–2012 (China)	62	32 (17/15, 58)	30 (19/11, 57)	8
Zhang [15]	2014	NRCT	2009–2013(China)	395	347 (271/73, 57)	48 (22/26, 58)	8
Xu [16]	2015	NRCT	2000–2013 (China)	407	206 (124/82, 57)	201 (128/73, 58)	7
Meng [17]	2015	NRCT	2009–2013 (China)	203	98 (57/41, 62)	105 (68/37, 60)	8
Watanabe [18]	2015	NRCT	2008–2013 (Japan)	185	98 (57/41, 67)	87 (47/40, 70)	8

*EI* enrolment interval, *BG* Braun group, *NBG* non-Braun group, *RCT* randomised controlled trial, *NRCT* non-RCT, *M* male, *F* female, ^a^years

#### Outcome assessment

The outcomes are shown in Table 2.

**Table 2:** Results of a meta-analysis

Outcome of interest	No. of studies	No.of patients	Results		OR/WMD	95% CI	*P*-value	*I*^2^ (%)
			Braun	Non-Braun				
Overall DGE	11	1650	11.5	26.6%	0.32	0.24, 0.43	<0.001	46
Clinically significant DGE	9	1558	7.7%	21.5%	0.27	0.15, 0.51	<0.001	55
Operative time (min)	8	1059	–	–	21.8	−3.37, 45.72	0.09	83
Estimated blood loss (mL)	9	1454	–	–	−55.15	−151.46, 41.15	0.26	90
Transfusion	6	575	20.8%	25.7%	0.73	0.49, 1.09	0.12	28
Vomiting	4	651	16.9%	34.1%	0.42	0.27, 0.65	<0.001	0
NT reinsertion	3	685	6.6%	12.9%	0.43	0.23, 0.81	0.009	0
Prokinetics or antiemetics	3	256	30.7%	39.8%	0.58	0.34, 1.00	0.05	0
Pancreatic fistula	11	1664	12.0%	19.1%	0.62	0.38, 1.02	0.06	50
Bile leak	9	1561	3.3%	5.5%	0.50	0.29, 0.86	0.01	0
Intra-abdominal abscess	6	851	10.9%	8.4%	0.80	0.46, 1.41	0.44	0
Wound infection	8	1069	11.8%	10.5%	0.99	0.64, 1.15	0.95	0
Gastrointestinal hemorrhage	6	1216	4.5%	4.3%	1.14	0.63, 2.08	0.67	0
Pneumonia	6	962	6.5%	7.1%	0.91	0.53, 1.58	0.25	44
Urinary tract infection	4	382	3.3%	5.7%	0.48	0.18, 1.24	0.13	0
Mortality	11	1672	1.4%	1.6%	0.75	0.34, 1.68	0.48	0
Length of hospital stay (days)	7	969	–	–	−1.66	−2.95, − 0.37	0.01	0

*DGE* delayed gastric emptying, *NT* nasogastric tube, *OR* odds ratio, *WMD* weighted mean difference, *CI* confidence interval

#### Primary outcomes

**Fig. 2:**
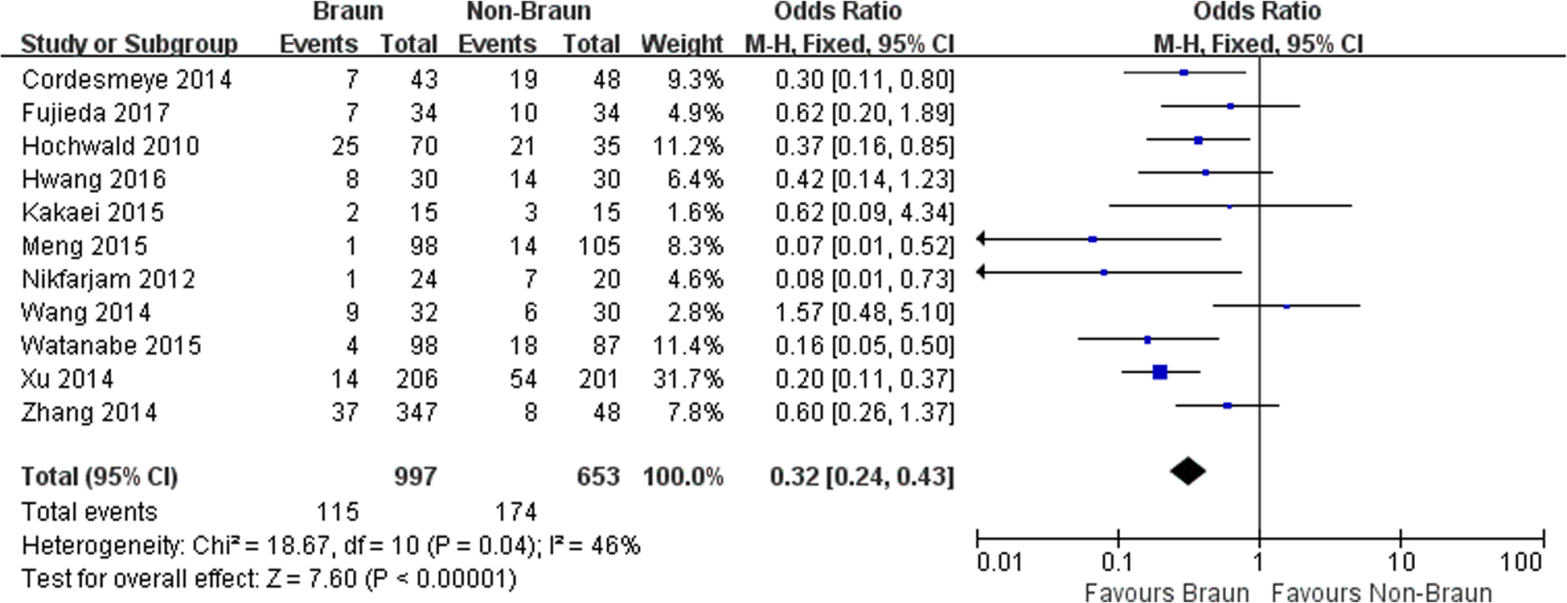
Results of the meta-analysis on delayed gastric emptying

**Fig. 3:**
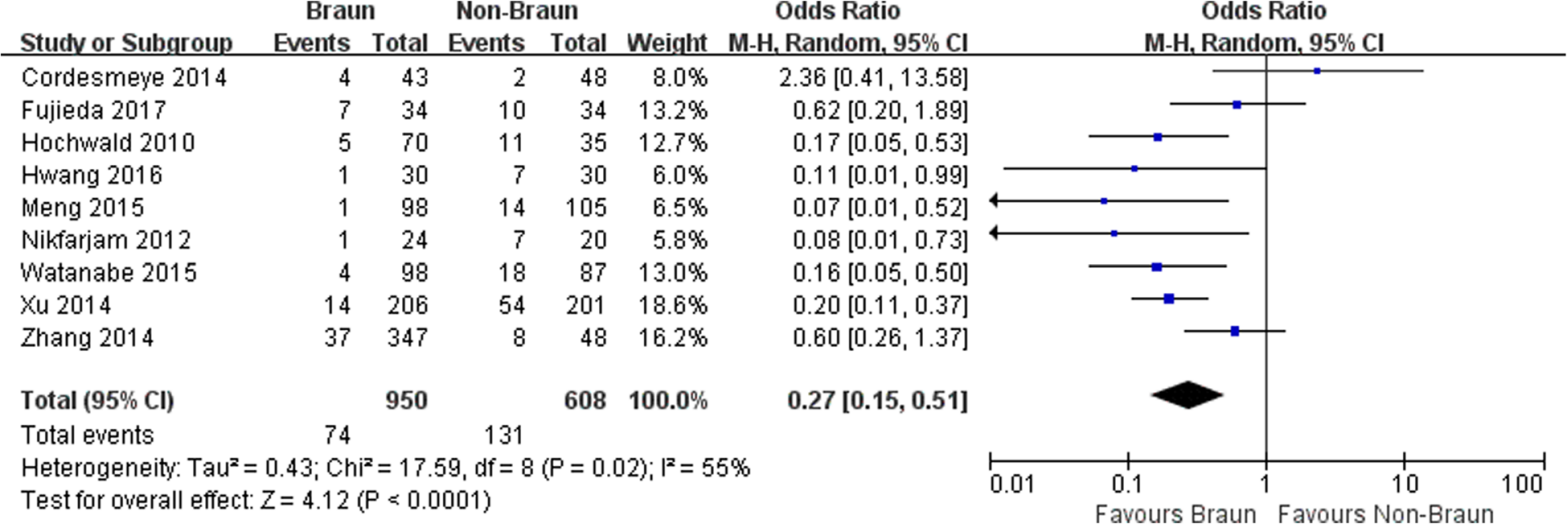
Results of the meta-analysis on clinically significant delayed gastric emptying

Both overall DGE and clinically significant DGE in Braun group were significantly lower than those in non-Braun group (OR 0.32, 95% CI 0.24 to 0.43 and OR 0.27, 95% CI 0.15 to 0.51, both *P* < 0.001) (Fig. 2-3). No complication directly attributable to Braun enteroenterostomy such as anastomotic leakage or bleeding was reported in any eligible study.

#### Secondary outcomes

Regarding operative details, no significant difference was observed in operative time, estimated blood loss, and requirement of blood transfusion between the two groups.

DGE-related clinical parameters were all significantly lower in the Braun group, including the incidence of vomiting (OR 0.42, 95% CI 0.27 to 0.65; *P* <0.001), nasogastric tube reinsertion (OR 0.43, 95% CI, 0.23 to 0.81; *P* = 0.009), and the use of prokinetics or antiemetics (OR 0.58, 95% CI, 0.34 to 1.00; *P* = 0.05).

In Braun group, the number of bile fistulas was significantly lower (OR 0.50, 95% CI, 0.29 to 0.86; *P* = 0.01). There was no significant difference in other postoperative complications and mortality. Following Braun enteroenterostomy, the length of hospital stay was estimated to be 1.66 days shorter than that for patients with no Braun enteroenterostomy (WMD -1.66, 95% CI, − 2.95 to − 0.37; *P* = 0.01).

#### Publication bias

**Fig. 4:**
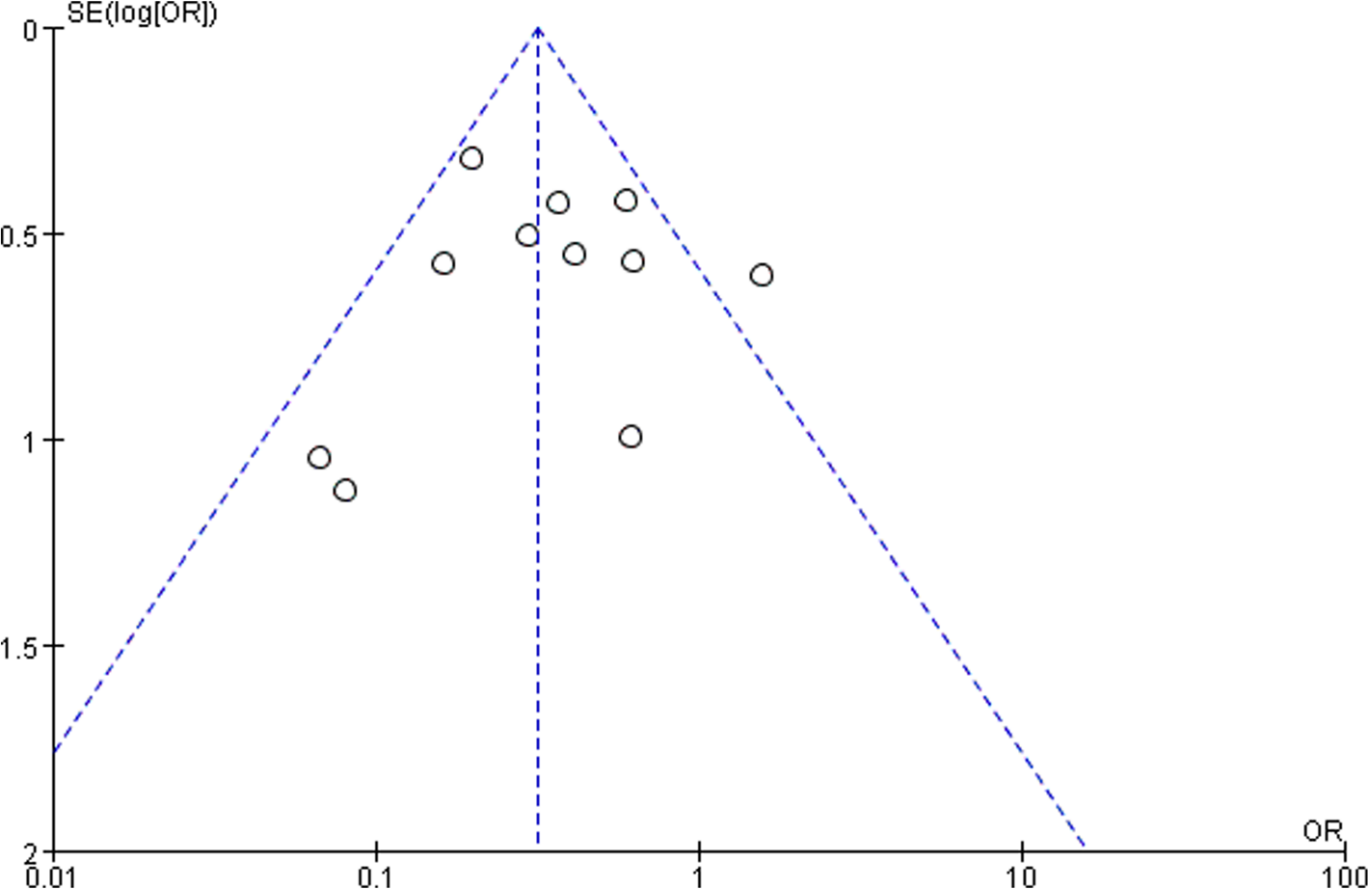
Funnel plot analysis of publication bias. The outcome was the overall delayed gastric emptying

Funnel plots based on the DGE is shown in Fig. 4. Only one study lay outside the limits of the 95% CI, indicating weak evidence of publication bias.

## Discussion

With refinements in surgical techniques and advancements in postoperative management, the mortality rate after pancreaticoduodenectomy has fallen below 5% in specialized centers around the world, but the morbidity rate remains high as 30–65% [19]. One major cause of morbidity is that DGE occurs at a frequency of 14–61% [1]. Risk factors for DGE include perioperative diabetes and postoperative complications such as pancreatic fistula [20]. Several surgical techniques for reducing the incidence of DGE have been attempted, including the type of pancreaticoduodenectomy (pylorus-resecting pancreaticoduodenectomy vs. pylorus-preserving pancreaticoduodenectomy) [1], the reconstruction type of gastrojejunostomy (Billroth II vs. Roux-en-Y) [21], and the route of gastro- or duodenojejunostomy (antecolic vs. retrocolic) [22]. Unfortunately, RCTs on these technical measures are scarce. As a result, there is no universal agreement regarding one particular variation being less prone to DGE than the others.

Braun enteroenterostomy was first reported about 100 years ago and has gained favor in recent years as a potential means to reduce the incidence of DGE following pancreaticoduodenectomy. Theoretically, Braun enteroenterostomy potentially stabilizes the afferent and efferent limbs of the gastrojejunostomy. The gastrojejunostomy itself becomes more stabilized, with a low tendency to twist and angulate [12]. In addition, Braun enteroenterostomy adequately diverts a substantial amount of bile from the afferent limb, thereby decreasing the likelihood of reflux gastritis. It also reduces tension on the anastomosis [11, 12]. However, there are limited studies on the effectiveness of Braun enteroenterostomy on DGE following pancreaticoduodenectomy and the results are conflicting [4–6, 11–18]. Meta-analysis provides a way to increase statistical power and resolve inconsistencies. Our pooling data have shown that Braun enteroenterostomy can reduce the rate and severity of DGE compared with non-Braun enteroenterostomy after pancreaticoduodenectomy. As expected, Braun enteroenterostomy also showed advantages in terms of clinical parameters related to DGE and length of hospital stay. These results are comparable to previous results from two earlier meta-analyses [2, 3]. The strength of the present study lies in its large number of patients.

In contrast to two previously published meta-analyses that found no significant difference in bile fistula between the two groups [2, 3], the present update has demonstrated a statistically significant association between Braun enteroenterostomy and a decreased rate of this complication. The incidence of pancreatic fistula also tended to be lower in Braun group with marginal statistical significance (*P* = 0.06). The weight of three recent RCTs seems important in these findings and participated to increase the measured magnitude of effect size [4–6]. It could be hypothesized that Braun enteroenterostomy can decrease biliopancreatic limb pressures, thus decreasing the risk of biliary and pancreatic fistula.

The present analysis has some limitations. First, significant statistical heterogeneity was detected between studies for some outcomes including the analysis of clinically significant DGE (*I*^2^ = 55%), largely due to the fact that there are significant variations in each clinical setting regarding surgical technique and perioperative care. Second, the level of evidence is low, for a considerable number of data came from NRCTs, knowing that NRCTs have inherent risk of bias. More larger-size RCTs are required to confirm our finding. Finally, long-term outcomes such as the nutritional status were not analyzed in this meta-analysis due to the limited data.

## Conclusions

The present meta-analysis shows that Braun anastomosis is associated with a less severe and lower incidence of DGE following pancreaticoduodenectomy.

## Abbreviations

95% CI: 95% confidence interval
DGE: Delayed gastric emptying
ISGPS: International Study Group of Pancreatic Surgery
NRCTs: Non-randomised controlled trials
NT: Nasogastric tube
OR: Odds ratio
PRISMA: Preferred reporting items for systematic reviews and meta-analyses
RCTs: Randomised controlled trials
WMD: Weighted mean difference
